# Imported Pediatric Lyme Disease in Singapore—A Case Series

**DOI:** 10.3390/pathogens15040437

**Published:** 2026-04-17

**Authors:** Ade Xin Ning Tan, Ilyas Hussin, Chia Yin Chong, Matthias Maiwald, Terri Xiao-Bei Chiong, Natalie Woon Hui Tan

**Affiliations:** 1 Department of Pediatrics, KK Women’s and Children’s Hospital, Singapore 229899, Singapore; ilyas.hussin@mohh.com.sg; 2 Infectious Disease Service, Department of Pediatrics, KK Women’s and Children’s Hospital, Singapore 229899, Singapore; chong.chia.yin@singhealth.com.sg (C.Y.C.); natalie.tan.w.h@singhealth.com.sg (N.W.H.T.); 3 Yong Loo Lin School of Medicine, National University of Singapore, Singapore 117597, Singapore; 4 Duke-National University of Singapore Medical School, Singapore 169857, Singapore; 5 Lee Kong Chian School of Medicine, Nanyang Technological University, Singapore 308232, Singapore; 6 Department of Pathology and Laboratory Medicine, KK Women’s and Children’s Hospital, Singapore 229899, Singapore; matthias.maiwald@singhealth.com.sg; 7 Department of Dermatology, KK Women’s and Children’s Hospital, Singapore 229899, Singapore; terri.chiong.x.b@singhealth.com.sg

**Keywords:** pediatric, Lyme disease, meningitis, prior antibiotic therapy

## Abstract

Lyme disease is the most common reported vector-borne disease in North America and is also highly prevalent across Europe. Although tick-borne diseases are uncommon in Singapore, there remains a risk of imported tick-borne diseases among travelers from endemic regions. We present a case series of three pediatric patients with imported Lyme disease managed at a tertiary children’s hospital in Singapore, illustrating the varied clinical presentations of Lyme disease in children. One child developed meningitis following prior antibiotic therapy for Lyme disease, although causality cannot be definitively established. This series aims to highlight key diagnostic considerations and management principles relevant to clinicians practicing in non-endemic regions.

## 1. Introduction

Lyme disease (LD) is the most common reported vector-borne disease in North America and is also highly prevalent across Europe [1,2]. It is a multisystem infection caused by spirochetes of the *Borrelia burgdorferi sensu lato* (Bbsl) complex and is transmitted to humans through the bite of infected *Ixodes* ticks [2]. The epidemiology and clinical manifestations of LD vary geographically. *Borrelia burgdorferi* sensu stricto predominates in North America but is also present in Europe, whereas *Borrelia garinii* and *Borrelia afzelii* are more commonly encountered in Europe and parts of Asia, and they are not present in North America [3].

The clinical syndrome of LD is classically divided into three stages (Early Localized or Stage I, Early Disseminated or Stage II, and Late or Stage III). Early localized infection most commonly presents with erythema migrans: a slowly expanding erythematous skin lesion at the site of a tick bite that typically develops 4 to 30 days after a tick bite [4]. If untreated, the infection may disseminate hematogenously, leading to a wide range of manifestations, including multiple skin lesions, Lyme neuroborreliosis (meningitis and cranial neuropathies), carditis, musculoskeletal, and ocular involvement [5,6]. Late disease may present with arthritis, chronic neurological syndromes, or acrodermatitis chronica atrophicans [6]. The Centers for Disease Control and Prevention (CDC) recommends a two-tiered serologic testing for LD [7].

Importantly, LD is generally highly treatable with appropriate antibiotic therapy, particularly when recognized in the early stages [4]. Delayed diagnosis may result in disseminated disease. However, neurological manifestations can occur even after initiation of antibiotic treatment in some cases of LD [8]. This highlights the need for continued clinical vigilance and appropriate follow-up.

Although tick-borne diseases are not endemic in Singapore, the country’s status as a major international travel hub places clinicians at risk of encountering imported cases [9]. Diagnosis may be challenging in this context due to low clinical suspicion, variable presentations, and limited familiarity with the disease.

In this study, we present a case series of three pediatric patients with imported LD managed at a tertiary children’s hospital in Singapore. These cases illustrate the diverse clinical manifestations of LD, provide a descriptive and educational overview of clinical presentations and highlight diagnostic considerations in a non-endemic setting, and emphasize the importance of recognizing disease progression even after initial therapy for LD.

## 2. Methods

We retrospectively reviewed three cases of imported pediatric LD encountered at KK Women’s and Children’s Hospital, Singapore, from 2013 to 2025. Data collected included clinical manifestations, clinical course, treatment and diagnostic investigations, comprising a strong clinical suggestion in the first case, and confirmatory test results for the second and third cases. This retrospective case series was approved by the Singhealth Centralized Institutional Review Board (CIRB 2018/2205, approved on 19 July 2018, and amended on 4 August 2025).

### 2.1. Case 1

**Figure 1:**
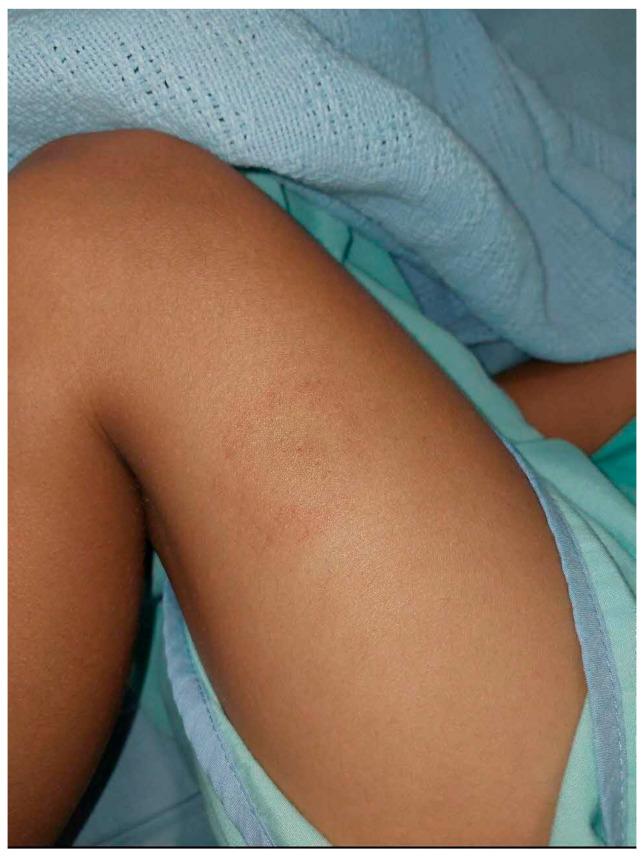
A solitary target-like patch over the left knee, with a central erythematous macule, a surrounding zone of clearing and an outer erythematous border, consistent with erythema migrans.

The first case describes a 7-year-old boy from Orange County, South California, USA, who presented with cutaneous LD while in Singapore en route to Manila, Philippines, for a sporting competition. He presented with a history of a solitary target-like patch over the left knee, with a central erythematous macule, a surrounding zone of clearing and an outer erythematous border, which expanded day by day over the course of 5 days, consistent with erythema migrans (Figure 1). The rash was first noted following outdoor playground activity. He was hemodynamically stable with no neurological or joint involvement.

Approximately one week prior to the onset of the rash, he had visited Crystal Cove beach in Orange County, California, where he had been catching sand crabs and sustained a presumed insect bite. He was also frequently exposed to outdoor environments, including playing in his home garden and with a neighbor’s dog. An electrocardiogram performed during admission was normal, with no evidence of cardiac conduction abnormalities. Dermatology was also consulted during the admission, with assessment of erythema migrans based on rash morphology and appearance. He was treated with a 14-day course of oral Cefuroxime, with good clinical recovery. Lyme serology obtained during admission subsequently returned negative. He subsequently returned to the USA for continued follow-up.

The presentation was clinically suggestive of early localized LD; however, in the context of uncertain exposure history with no supportive serological confirmation, diagnostic uncertainty remains, and alternative diagnoses, such as a hypersensitivity reaction, cannot be fully excluded.

### 2.2. Case 2

The second case describes cutaneous LD in a 1-year-10-month-old boy from Sweden. He presented with a 3-week history of erythema migrans over the left arm, with a central punctum suggestive of a tick bite, although no definite history of tick attachment was recalled. This was associated with a 3–4-day history of fever, with no neurological symptoms or joint involvement.

The rash developed approximately one month after a hiking trip to the wooded areas in the Swedish countryside near Stockholm. An electrocardiogram performed during admission showed no evidence of cardiac conduction abnormalities.

Lyme serology obtained 6–7 weeks after hiking exposure demonstrated positive Borrelia IgG and negative IgM. Further confirmatory testing for European Borrelia species, performed in Germany, showed elevated IgG levels and negative IgM on both ELISA and immunoblot assays, with positive IgG on immunoblot. These findings were consistent with LD. He was treated with a 14-day course of oral Cefuroxime, with good clinical recovery thereafter.

### 2.3. Case 3

The third case describes Lyme neuroborreliosis in a 7-year-old girl from Sweden. She presented to our hospital in Singapore with a 1-day history of acute right facial weakness, associated with a 2-day history of fever and headache. She had a documented history of a tick bite over her left forehead approximately 10 days prior to symptom onset while hiking in the wooded areas near Stockholm, Sweden. She was initially evaluated at a hospital in Sweden after the tick bite. She had not yet developed any facial weakness, had no other symptoms, and did not receive antibiotic treatment at that time.

Initial Lyme serology (Borrelia immunofluorescence assay) conducted in Singapore demonstrated positive IgM and IgG antibodies. She remained clinically stable during inpatient monitoring, with no evidence of cardiac involvement or additional neurological deficits and she was treated with a two-week course of oral Amoxicillin.

Two weeks later, she was readmitted to our hospital with features of meningitis and persistent right lower motor neuron facial nerve palsy. Cerebrospinal fluid analysis demonstrated mild pleocytosis with a white cell count of 60 cells/µL. Confirmatory Lyme serological testing showed a positive IgM immunoblot and a positive IgG immunoblot. She was subsequently treated with a 14-day course of parenteral Ceftriaxone for Lyme neuroborreliosis with facial nerve palsy. Her facial palsy resolved completely, and she had no further manifestations of LD on follow-up.

## 3. Discussion

The clinical phenotype of LD varies according to disease stage, host factors, and infecting Borrelia species, with notable geographical differences between North America and Europe. These variations contribute to diagnostic challenges, particularly in pediatric patients and in non-endemic regions. In Europe in particular, *Borrelia afzelii*, which is not present in North America, can cause late-stage acrodermatitis chronica atrophicans (ACA), a rare manifestation in pediatric age [10]. *Borrelia burgdorferi* sensu stricto is the predominant species in the USA, and *Borrelia mayonii* has also been isolated, which is characterized by being spirochaetemic [11]. The clear prevalence of *Borrelia burgdorferi* sensu stricto in the USA and at least seven Bbsl species in Europe also may contribute to differences in serological test performance and interpretation [12]. Given the small number of cases, this case series is intended to be descriptive and educational, highlighting diagnostic considerations in a non-endemic setting, rather than supporting broader clinical inferences.

### 3.1. Cutaneous Manifestations

Skin involvement may be observed in all three stages of LD.

Erythema migrans (EMs), formerly called erythema chronicum migrans of Afzelius-Lipschutz [13], from which the name *Borrelia afzelii* also derives, as well as Erythema chronicum migrans [14], represent the localized early phase of LD. EM occurs in 75% of cases 4 to 30 days after a tick bite (incubation time) and starts as an erythematous macule at the site of the tick bite, which expands centrifugally over the course of days or weeks to form a large erythematous plaque, usually measuring at least 5 cm in diameter, which can reach 30–40 cm. The classic bull’s eye appearance [4] only appears in the minority of LD cases. If left untreated, EM may persist for weeks, and spontaneous resolution may occur within a few weeks, but this does not necessarily mean resolution of the infection. The detection of EM after a tick bite in an endemic area, even with negative serology (pre-serological phase), is sufficient for the diagnosis of LD. Conversely, local hypersensitivity reactions to tick bites develop within a few hours and resolve within a few hours [4].

In the early disseminated phase, multiple erythemas are observed (secondary EM without signs of the tick bite in the center), which are less extensive and edematous than the primary lesion and may present atypical morphologies.

Stage II also includes Borrelial Lymphocytoma, which is most often located in the earlobe or mammary areola. Diagnosis is made by histological examination and immunophenotype (CD20+, Bcl2-); PCR for Bbsl can also be performed on DNA extracted from the tissue [15].

ACA is a late-stage manifestation caused by *Borrelia afzelii,* reported in European infections [16].

In our case series, the first two patients presented with EM, consistent with early localized disease. In Case 1, although the rash was clinically suggestive of erythema migrans, the absence of confirmatory laboratory findings and an uncertain exposure history necessitate a cautious interpretation. The recognition of EM in non-endemic settings is critical, as early serological testing may be negative and diagnosis remains largely clinical.

### 3.2. Musculoskeletal Involvement

It is typically asymmetric and can be monoarticular or oligoarticular. Episodes may be intermittent and migratory in the early disseminated phase, involving large joints, including the temporomandibular joint, while they are persistent in the late phase (Lyme arthritis). Unlike septic arthritis, Lyme arthritis is more often localized to the knees, is less painful, and is usually not associated with fever [4].

Appropriate antimicrobial therapy results in eradication of Borrelia infection and resolution of arthritis in most cases. However, a subset of patients may develop persistent synovitis lasting months to years, thought to be immune-mediated, and may require anti-inflammatory or disease-modifying therapy [4].

### 3.3. Cardiac Manifestations

Lyme carditis is an early disseminated manifestation that typically occurs 2–5 weeks after the onset of EM, although earlier presentations have been described. It most commonly manifests as atrioventricular conduction abnormalities of varying degrees. Patients may present with palpitations, presyncope or syncope, dyspnea, or chest pain. Rare manifestations include myocarditis and pericarditis [17].

In all three cases in this series, electrocardiographic screening revealed no evidence of cardiac involvement, underscoring the importance of routine cardiac assessment even in the absence of symptoms.

### 3.4. Lyme Neuroborreliosis

Lyme neuroborreliosis (LNB) occurs in approximately 10–15% of untreated patients, with the risk significantly reduced by early and appropriate antimicrobial therapy, although there are cases where LNB has occurred after EM treatment has started [18]. Early LNB typically presents days to weeks after tick exposure and may include lymphocytic meningitis, cranial neuritis, most commonly facial nerve palsy, radiculoneuritis, or encephalitis. Late neurological manifestations include polyneuropathy, which may be associated with ACA [8], chronic meningitis, encephalomyelitis, and, rarely, cerebrovascular complications related to vasculitis or stroke [19].

Our third case illustrates a typical presentation of early LNB with facial nerve palsy and meningitis following a documented tick bite in an endemic region, highlighting the importance of maintaining diagnostic vigilance even when patients present outside endemic areas. Notably, this case describes the development of meningitis following prior antibiotic therapy; however, this observation should be interpreted cautiously, as it may reflect the timing of treatment or natural disease progression.

## 4. Conclusions

To date, there have been no reported cases of locally acquired LD in Singapore. Nevertheless, clinicians should remain alert to the possibility of imported LD in children presenting with compatible clinical features, particularly in the context of recent travel to endemic regions.

This case series is descriptive in nature and aims to provide educational insights regarding the broad clinical spectrum of pediatric LD, ranging from early localized disease to neuroborreliosis, and underscores the diagnostic challenges in non-endemic settings where clinical suspicion may be low. Early recognition of characteristic features, especially erythema migrans, is essential, as prompt antibiotic treatment is highly effective and can prevent disease progression [4].

Importantly, our findings also emphasize that neurological manifestations may develop or evolve in some cases following initial antibiotic therapy, although this observation is based on a limited number of cases. Patients and caregivers should be advised regarding the potential for symptom progression and the need for ongoing monitoring and timely re-evaluation if new neurological or systemic features arise after antibiotic therapy for LD [20].

## Data Availability

The data presented in this study are available on request from the corresponding author due to patient privacy.

## Abbreviations

The following abbreviations are used in this manuscript:

LD	Lyme Disease
CDC	Centers for Disease Control and Prevention
ELISA	Enzyme-linked immunosorbent assay
EM	Erythema migrans
ACA	Acrodermatitis chronica atrophicans
LNB	Lyme neuroborreliosis
